# One for Everyone: A Study of User Satisfaction Among Health-Care Providers Regarding Extended Use of N95 Masks During the COVID-19 Pandemic

**DOI:** 10.1017/dmp.2020.380

**Published:** 2020-10-12

**Authors:** Nishant Sharma, Anant Gupta, Makhdoom Killedar, Ashish Bindra, Asmita Patil, Surabhi Gupta, Paavan Gopathoti, Parmeshwar Kumar

**Affiliations:** Department of Hospital Administration, AIIMS, New Delhi, India; Department of Neuroanaesthesiology and Critical Care, AIIMS, New Delhi, India; Department of Physiology, AIIMS, New Delhi, India; Department of Reproductive Biology, AIIMS, New Delhi, India

**Keywords:** COVID-19, extended use, health-care providers, N95 masks, pandemic, respiratory protection

## Abstract

**Objective:**

This study was conducted to assess the feasibility of extended use of N95 masks in our hospital during the coronavirus disease 2019 (COVID-19) pandemic. We also studied the use pattern, user satisfaction, and issues faced during extended use of the mask.

**Methods:**

This cross-sectional study was conducted among health-care providers in a large tertiary care teaching hospital in northern India from April 1 to May 31, 2020. A list was prepared from the institute’s register, and participants were chosen by random sampling. The data collected from the physical forms were transferred to excel sheets.

**Results:**

A total of 1121 responses were received. The most common problem stated with reuse of N95 masks was loss of fit followed by damage to the slings, highlighted by 44.6% and 44.4% of the participants, respectively. A total of 476 (42.5%) participants responded that they would prefer “cup-shaped N95 mask with respirator”. The median scores regarding the satisfaction with the quality of masks and their fit was also 4 each.

**Conclusions:**

It was concluded that the extended use of N95 masks was acceptable, with more than 96% of the participants using these masks.

The burden of coronavirus disease 2019 (COVID-19) pandemic is likely to be with us for some time, as the virus continues to wreak havoc on health-care infrastructures and economies and has spread quickly across countries as measures such as lockdowns ease. While development of vaccines remains in the trial phase and definitive treatment remains elusive, the nonpharmacological interventions, such as personal hygiene, social distancing, and the use of masks, are the only measures for preventing or slowing disease transmission. For health-care professionals (HCPs), masks remain the cornerstone for personal protection as these HCPs provide medical care to COVID patients at close quarters.^1^

The temporal pattern and incident patient load or burden of the disease remains unpredictable during pandemics because of the various regional, cultural, and behavioral determinants of the affected population.^2^ Due to lack of adequate time for stockpiling and speeding up production, countries face supply shortages of respirators, or medical masks, for the HCPs. In fact, trepidations are emergent over global shortages of respiratory masks during the current COVID-19 pandemic and strategies for optimizing their availability to HCPs.^3^ Solutions to the above are make-shift masks; extended use and/or reuse of available N95 masks may have limited credibility.^4,5^ The idea behind extended use and/or reuse of disposable N95 respirators and masks is to offer a level of protection beyond their intended limits of use.^5,6^

According to CDC guidelines, reuse of mask is defined as a HCP donning the same mask for a series of close patient contacts and doffing it at the end of each of the close patient contacts before it is discarded.^7^ There are stringent regulations for mask reuse, and the mask has to be discarded if it is contaminated or damaged. Hence, this reuse is referred to as “limited mask reuse.” This N95 respirator “limited reuse” has been recommended and widely used as an option for conserving respirators during previous respiratory pathogen outbreaks and pandemics.^8,9^ Guidelines for limited mask reuse were established when they were first introduced for HCPs in close contact with tuberculosis patients.^10^

In the present study, the N95 masks were issued as personal protection for HCPs, in addition to the designated COVID areas where reuse masks were used. These were designated as “personal” use N95 masks to be used in common circulation areas, offices, etc., within the hospital setting.

This study was conducted to devise a strategy for implementation of CDC guidelines for reuse/extended use of N95 masks in a tertiary care hospital during the COVID-19 pandemic. We also studied the use pattern, user satisfaction, and issues faced by HCPs during extended use of the mask using a questionnaire.

## METHODS

A cross-sectional study was conducted in a large tertiary care teaching hospital in New Delhi, and all the employees who were willing to participate were included in the study. The study period was from April 1 to May 31, 2020.

The decision to implement the CDC guidelines for reuse/extended use of N95 masks was taken in April 2020 by the institute administration, and it was decided to issue a set of 5 N95 masks to all HCPs for a period of 20 d. Each mask was supplied in a paper envelope, which was used to store the mask following each use.

During the rollout, a multi-pronged approach was adopted for training the HCPs regarding reuse/extended use of these N95 masks; after the first cycle, feedback was sought from the HCPs regarding the most suitable method for spreading awareness regarding the appropriate use of these masks. A structured questionnaire was developed containing 17 questions. The questionnaire consisted of 4 sections. The first section contained 4 questions that had the demographic details of the participants. The second section contained 6 questions related to use of the N95 masks, the third section contained 3 questions related to the fit, and fourth section had 4 questions related to the overall satisfaction with the use of N95 masks. The questionnaire was bilingual (Hindi and English) and was created in both physical print form and an electronic version in Google forms. The questionnaire items were pooled and reviewed by a group of experts from different specialties. It was ensured that the items were free from any construction and semantic problems, grammatical errors, and it was ensured that it was easy to comprehend by varied strata of HCPs.

Sample size was calculated to be 1250 taking power as 80%, alpha 0.05, precision as 3%, nonresponse rate of 10% and prevalence as 50% (no previous similar study, so assumption). A list was obtained from the institute of all the health-care workers (HCWs). The sample was taken using random number table generated by computer. A link of the Questionnaire in Google Forms was circulated electronically to the members of the list through various channels, including groups on different social media platforms, using appropriate software, Facebook and WhatsApp applications. The physical questionnaire was distributed to the Sanitation Staff, Security Guards, Medical Records Staff, Stores Department, Engineering Maintenance Staff, Accounts Staff, and other administrative staff and was collected back after a week. Data were then transferred into Microsoft Excel 2016. Data cleaning was done in Microsoft Excel 2016. Analysis was done in Stata 11 (StataCorp, College Station, TX). Data were analyzed using descriptive statistics and have been presented as proportions while bivariable analysis between 2 categorical variables was done using chi-squared test, and *P*-value less than 0.05 was considered significant.

## RESULTS

A total of 1121 responses were received during the data collection of the study at a response rate of 89.7%. Among the participants, 638 (56.9%) were male and 483 (43.1%) were female. The mean age of participants was 36.4 years.

The majority of the participants were Nursing Officers (29.4%), followed by Security personnel (27.6%). Responses were also received from Faculty, Resident Doctors, Administrative Nurses, General Administrative Officials, Technical Staff, and Sanitation and Housekeeping workers of the Institute.

A total of 911 (81.3%) participants stated that they had received the N95 masks for extended use along with brown covers. However, 200 (17.8%) received only the masks, while 8 (0.7%) claimed that they had not received the masks or their covers (Table 1).

**TABLE 1:** Demographic Details of the Participants

Questions	Categories	No. of Participants	Percentage (%)
Gender	Male	638	56.9
	Female	483	43.1
Profession/cadre	Nursing officers	330	29.44
	Security personnel	309	27.56
	Sanitation staff	160	14.27
	Resident doctors	152	13.56
	Faculty	121	10.79
	Technical staff	29	2.59
	Administrative nursing cadre (NS/ DNS/ ANS)	11	0.98
	General administration officials	09	0.80
Received the masks and brown envelope	Mask and brown envelope	911	81.3
	Masks only	200	17.8
	Envelopes only	2	0.2
	Nothing	8	0.7
Age	Mean (y)	36.35	
	Maximum (y)	20	
	Minimum (y)	72	

The decision to implement the CDC guidelines for reuse/extended use of N95 masks was taken by the Institute administration based on the recommendation of the Hospital Infection Control Committee in April 2020, and it was decided to issue a set of 5 N95 masks to all HCPs for a period of 20 d. Based on the guidelines, the masks were to be rotated daily and would be used for 5 cycles, each, at an interval of 4 d. The initial challenge was to educate the HCPs regarding appropriate use of these masks. During the rollout, a multi-pronged approach was adopted for training the HCPs regarding reuse/extended use of these N95 masks. An official circular was drafted and circulated throughout the institute as a hard copy as well as through email. The masks were distributed in brown envelopes, each of which contained 5 N95 masks and 4 small brown envelopes for storage. A bilingual video (Hindi & English) was also prepared and circulated on various social media platforms within the Institute.

Feedback was sought from the HCPs regarding the most suitable method for spreading awareness regarding the appropriate use of these masks. According to the participants, “pasting instructions on the brown paper covers” was the best method of passing information regarding the use of these masks. This was suggested by 482 (43%) of the participants. The other preferred methods suggested were circulation of hard copy of official order (25.5%) and circulation of video regarding use on social media (23.6%).

A total of 1082 (96.5%) participants used the set of N95 masks issued to them by the hospital. However, 17 (1.5%) used personally purchased N95 masks, while 22 (2%) stated that did not use any mask during the study period. Among the clinical staff, 2.69% did not use the N95 masks provided to them while among the nonclinical staff; only 0.20% did not use them. This difference was statistically significant with a *P*-value of 0.001.

The majority of the participants, 459 of 1121 (40.9%), responded that they used the masks for more than 15 d during the 20-d use cycle, while 269 (24%) had used them for 10-15 d, 177 (15.8%) for 5-10 d, and 216 (19.3%) for less than 5 d.

A total of 1038 (92.6%) participants used the masks during duty hours at the hospital. However, 483 (43.1%) used them during their travel to and from, 163 (14.5%) also used them during their visits to buy essential commodities, while 44 (3.9%) used them only while doing procedures on patients.

Most of the employees, 801 (71.5%), said that they used the masks for more than 6 h/d, while 226 (20.2%) used them for 4-6 h, 28 (2.5%) used the mask for less than 2 h (Table 2),

**TABLE 2:** Utilization Pattern of N95 Masks Provided for Extended Use

Questions	Categories	No. of Responses	Percentage (%)
Use of masks issued by the hospital	Yes	1082	96.5
	No	39	3.5
Number of days mask was used	10-15 d	269	24.0
	5-10 d	177	15.8
	less than 5 d	216	19.3
	more than 15 d	459	41.0
Number of hours mask was used each day	2-4 h	66	
	4-6 h	226	20.2
	less than 2 h	28	2.5
	more than 6 h	801	71.5
Place of use	During duty hours at hospital	1038	92.6
	During travel to and from hospital	483	43.1
	During your visit to buy essential commodities	163	14.5
	Only while doing procedures on patients	44	3.9

The masks were a proper fit for 79.4% of the participants, while the rest stated that the masks did not fit them properly.

The most common problem stated with reuse of N95 masks was loss of fit followed by damage to the slings, doubt regarding the effectiveness and bad odor, highlighted by 44.6%, 44.4%, 41.6%, and 18.5% of the participants, respectively. Among the female participants, 22.62% stated that there was a problem of bad odor while among males 13.7% stated it as a problem. This difference was statistically significant with a *P*-value of 0.006.

A total of 476 (42.5%) participants responded that they would prefer “cup-shaped/fixed shape N95 masks with respirator,” if given a choice. The “duckbilled masks” were preferred by 24.5%, “masks with ear slings and respirator” was preferred by 18.8%, and “KN95 masks” by 12.1%.

**FIGURE 1:**
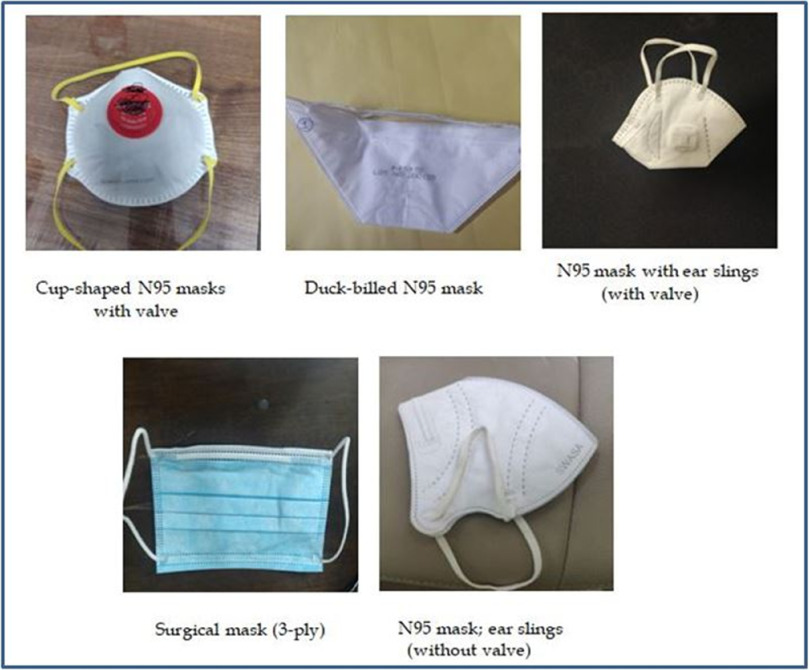
Various Types (Size and Shape) of N95 Masks Provided to HCPs.

The cup-shaped N95 masks with respirator were preferred by 45.54% of the males and 38.3% of the females, the ear-sling with respirator was preferred more among the females (22.98%) as compared to males (15.49%), the duck-billed were preferred by 20.5% of the females and 27.86% of the males. The difference in preference of masks among both the genders was statistically significant with a *P*-value of <0.001. There was also a difference in the preferred type of masks among the clinical and nonclinical staff. Among the clinical staff, the preferred type of mask was cup-shaped with respirator (40.81%), ear-sling with respirator (28.78%), duck-billed (16.59%), KN95 (10.24%), and 3-ply surgical masks (3.58%). The nonclinical staff preferred cup-shaped masks with respirator (44.38%) followed by duck-billed (34.52%), KN95 (14.20%), ear-sling with respirator (6.51%), and 3-ply surgical masks (0.34%). This difference in preference of masks among clinical and nonclinical staff was also significant with a *P*-value of <0.001 (Figure 1).

The fourth section of the questionnaire required the participants to rate their satisfaction regarding various parameters on a scale of 1 to 5, 1 being the least satisfactory and 5 being the most. The median scores regarding the satisfaction with the quality of masks and their fit was also 4 each.

The satisfaction regarding the quality was found to be significantly higher among the nonclinical staff (84.62%) as compared to the clinical staff (39.38%) and among males (65.05%) as compared to female participants (52.80%). The *P*-value for both was <0.001. The satisfaction regarding quality was higher among the participants who stated that they used the masks for a longer duration. Among the participants who used the masks for more than 6 h, 62.05% were satisfied with the quality while 48.39% participants who used the masks for less than 2 h were satisfied. The *P*-value was 0.014.

Similarly, the satisfaction regarding the fit was significantly higher among the nonclinical staff (85.6%) as compared to the clinical staff (41.98%). There was a statistically significant difference between the satisfaction level regarding the fit of the masks among male (66.46%) and female participants (55.28%); the *P*-value being <0.001. The satisfaction regarding fit was higher among the participants who stated that they used the masks for more than 6 h, 61.65% as compared to those who used it for lesser duration, 59.73% among those who used them for 4-6 h, 46.97% among those who used them for 2-4 h, and 54.84% for those who used the masks for less than 2 h. The *P*-value was 0.039 (Table 3).

**TABLE 3:** Comparison of Satisfaction With Quality and Fit of N95 Masks Among Various Categories

Questions	Categories	Percentage of Participants Who Were Satisfied	*P*-Value Using Chi-Squared
Satisfied with quality of N95 masks	Clinical staff	39.38	<0.001
	Non clinical staff	84.62	
	Males	65.05	<0.001
	Females	52.80	
	Used for <2 h/d	48.39	0.014
	Used 2-4 h/d	43.94	
	Used 4-6 h/d	57.96	
	Used >6 h/d	62.05	
Satisfied with fit of N95 masks	Clinical staff	41.98	<0.001
	Non-clinical staff	85.60	
	Males	66.46	<0.001
	Females	55.28	
	Used for <2 h/d	54.84	0.039
	Used 2-4 h/d	46.97	
	Used 4-6 h/d	59.73	
	Used >6 h/d	61.65	

## DISCUSSION

Because coronaviruses lose their viability significantly after 72 h, a rotation and re-use can be done assuming there is no soiling and minimal to no viral contamination of the mask.^11^ Studies suggest that the protection margin of limited reuse N95 masks begins to decrease after multiple donning, which varies with the ambient conditions and individual use. The type of N95 mask, ie, duck-billed or dome shape and its fit on the individual’s face also affected the duration of extended and limited use.^12^ Hence, it is preferable to limit the number of reuses to 5 to warrant an adequate safety margin.^7^ The CDC suggests that 5 masks to be acquired for 20 d and rotated daily, allowing them to dry for at least 72 h. In these interim periods, the masks are to be air dried by hanging or placement in individual, clean, breathable container like a paper bag.^13,14^

In conclusion, the effectiveness of N95 masks depends on fit, level of exposures, and appropriate use. Moreover, the mask would only protect from oro-nasal aerosol contamination and not against contact transmission.^15,16^ Despite the above limitations, these reused masks provided better personal protection in comparison to cotton masks and homemade alternatives, such as handkerchiefs, or no protection at all.^13^ Still, extended use and re-use of single-use masks/respirators should only be considered in situations of critical shortage. Also, when practicing extended use or re-use, policies have to be fixed and individuals made aware of these guidelines.^17^ There is paucity in the literature of such clear and consistent guidelines, which warrants further attention. After due consideration of all the factors, the decision to provide N95 masks for extended use for all employees was taken, as was suggested by a 2400-bed hospital in Seoul that emphasized on respiratory protection to all patients and employees.^18^ In a communication by Jeon et al. on the experience of protection of HCWs against COVID-19 at a large teaching hospital in Seoul, there was evidence of marked reduction in risk to HCWs from category 3 (needing self-quarantine) to category 0 (usual management) with the use of 4 personal protective equipment (PPE) items, which included N95 respirators.^18^

Patel et al. suggested that, during the 2009 H1N1 pandemic, projecting the demand and supply of N95 respirators was a major challenge, because the pandemic as well as the supply chain were unpredictable.^19,20^ They also suggested that extended use or reuse of N95 masks was sometimes considered by HCWs to tide over the crisis.^19,21^ The institution anticipated the same challenges when it decided to implement extended use of N95 masks rather than using them for just 1 time.

Tan et al. studied the effect of extended use of N95 respirators on PPE use in COVID-19 outbreak wards of a Singapore hospital. After implementation of extended use, the average use of N95 respirators dropped down to 1710 from 2490.^22,23^ They further documented that, after nearly 45 d of implementation, there was no confirmed case of severe acute respiratory syndrome coronavirus 2 (SARS-CoV-2) infection among the HCWs who had acquired the same from the hospital. The N95 masks were also found not to be contaminated after patient contact.^22,24,25^

In 2020, Garcia Godoy et al. conducted a review regarding facial protection of HCWs during pandemics. They concluded that among HCWs, the rates of respiratory infections were less with use of N95 masks as compared to surgical masks.^26-29^ They also stated that extended use of N95 masks along with other infection control measures, such as hand hygiene poses minimal risk to the HCWs.^23,26,30^ The literature also suggests that continuous use of N95 masks was better as compared to sporadic use at the time of high-risk procedures,^26,27^ further strengthening our decision to provide N95 masks to all the employees.

Conservation, extended use, and reuse after decontamination have been described as PPE supply strategies in view of increased demand and failing supply line by Fillingham et al.^30-34^

The most preferred method about disseminating information regarding the use of this mask was pasting instructions on the brown envelopes in which the masks were supplied.

The overall use of N95 masks provided for extended use was 96.5%, and most of the participants used these masks during their entire stay in the hospital. This is imperative for preventing the transmission of infection from asymptomatic or mildly symptomatic HCPs to other providers and patients^35^ as the study by Hu et al. suggests that unprotected exposures are more likely from patients who did not have influenza-like symptoms.^36,37^ The nonuse of masks provided for extended use was more among the clinical staff as compared to the nonclinical staff. This might be due to the fact that the clinical staff has access to other sources of masks, such as inpatient areas, outpatient departments, and/or procedure rooms, where separate masks were provided for front-line HCWs.

The 5 masks were provided for use during a 20-d cycle, after which they would again be reissued. Although the majority of the participants were satisfied with the quality and fit of the masks, 60% of them did not use it for more than 15 d. Another probable reason for this could be a problem with the N95 masks, which were loss of fit and damage to the slings, as highlighted by 44.6% and 44.4% of the participants, respectively. Also, it was observed that most of the participants used these masks for more than 6 h/d, which was probably because they used them during the duty hours (92.6%) and the shift are usually 8 h long.

In our study, the participants stated that they preferred the cup-shaped mask the most, followed by the duck-billed ones, and lastly the N95 masks and KN95 masks. The literature suggests that the cup-shaped and duck-billed masks are easy to don and doff and are more comfortable as compared to particulate respirators (N95, KN95, FFP2, FFP3, etc.)^38-40^ Also, in our study, surgical masks were considered to provide adequate protection against COVID-19 only by 3.58% of the clinical staff and 0.3% of the nonclinical staff. This finding is consistent with the findings of the study conducted by Hu et al. among HCWs in 21 ICUs in China during the 2009 H1N1 pandemic, where it was revealed that 33.1% of the respondents believed that surgical masks provide adequate protection as opposed to 88.3% who believed N95 masks to be adequate.^36^

The satisfaction regarding the quality and fit of N95 masks was higher among the nonclinical staff as compared to the clinical staff as the latter has to use the masks continuously for a longer duration as they worked in high-intensity patient care areas. Another reason for dissatisfaction among the clinical staff could be that they had the option to use other types of N95 masks supplied to the clinical area, which were single-use ones. The participants who stated that they used the masks for a longer duration were more satisfied with the quality and fit of the masks, thus reinforcing that good quality and fit is necessary for better compliance.

A study conducted by Scarano et al. regarding the effects of protective face masks on skin temperature and comfort revealed that use of N95 masks was associated with raised skin temperature in the facial region leading to greater discomfort as compared to surgical masks.^41^ Another study by Hua et al. on short-term effects of use of N95 masks, states that skin reaction were more common with N95 masks as compared to surgical masks.^42^ However, these studies were conducted on subjects in nonclinical setting where the fear of transmission of infection is eliminated. However, in our study, the participants were involved in thecare of suspected and confirmed COVID-19 patients and probably the prevention of transmission was a priority over the discomfort associated with use of N95 masks. The result of our study can be interpreted accordingly.

In a study by Rebmann et al. on effects and compliance of N95 mask use among Nursing staff of ICUs, it was concluded that there was no clinically significant physiological burden for health-care personnel apart from certain subjective symptoms, and the compliance was fairly high, despite the discomfort and repeated readjustments, which increased over time.^43^ In our study, compliance and satisfaction were found to be high as the survey was conducted during early phases of the pandemic when the fear among general population and the health-care personnel was high.

The limitation of the study was that the survey was carried out through a structured questionnaire, which may have tempted the participants to answer the most acceptable response rather than what is their true perception, and it was carried out in a very short period of time, as the future course of action regarding the supply of these masks had to be decided.

## CONCLUSIONS

It was concluded that the extended use of N95 masks was acceptable, with more than 96% of the participants using these masks. Most of them used the masks during their duty hours (92.6%) and the duration of use was more than 6 h for 71.5% of the participants. Out of the stipulated 20-d period during each cycle, the masks were used for more than 15 d by majority of the respondents (40.9%). According to the participants, the best method to disseminate information regarding the extended use of these masks was by pasting instructions on the brown envelope. The authors used a multi-pronged approach for passing on information to the users. The majority of the participants preferred the cup-shaped/fixed-shape N95 masks with front valve over other types of masks. The maximum participants rated their satisfaction with the fit and quality of N95 masks as 4 on a scale of 1 to 5. The participants who were more satisfied used the masks for longer duration.

Because the majority of participants were satisfied with the extended use of N95 masks, there are definite advantages, not only in financial terms but also to preserve a precious and scarce resource at the time when the demand outnumbers the supply. However, it is recommended to study the effect of extended use of N95 masks on the risk of transmission of respiratory infections to HCPs.
